# RNA-seq and GSEA identifies suppression of ligand-gated chloride efflux channels as the major gene pathway contributing to form deprivation myopia

**DOI:** 10.1038/s41598-021-84338-y

**Published:** 2021-03-05

**Authors:** Loretta Giummarra Vocale, Sheila Crewther, Nina Riddell, Nathan E. Hall, Melanie Murphy, David Crewther

**Affiliations:** 1 Department of Psychology and Counselling, La Trobe University, Melbourne, VIC Australia; 2 Department of Animal, Plant and Soil Sciences, La Trobe University, Melbourne, VIC Australia; 3 Centre for Human Psychopharmacology, Swinburne University of Technology, Melbourne, VIC Australia; 4 School of Health and Biomedical Sciences, RMIT, Melbourne, VIC Australia

**Keywords:** Gene expression, Transcriptomics, Ion channels in the nervous system, Molecular neuroscience, Visual system

## Abstract

Currently there is no consensus regarding the aetiology of the excessive ocular volume that characterizes high myopia. Thus, we aimed to test whether the gene pathways identified by gene set enrichment analysis of RNA-seq transcriptomics refutes the predictions of the Retinal Ion Driven Efflux (RIDE) hypothesis when applied to the induction of form-deprivation myopia (FDM) and subsequent recovery (post-occluder removal). We found that the induction of profound FDM led to significant suppression in the ligand-gated chloride ion channel transport pathway via suppression of glycine, GABA_A_ and GABA_C_ ionotropic receptors. Post-occluder removal for short term recovery from FDM of 6 h and 24 h, induced significant upregulation of the gene families linked to cone receptor phototransduction, mitochondrial energy, and complement pathways. These findings support a model of form deprivation myopia as a Cl^−^ ion driven adaptive fluid response to the modulation of the visual signal cascade by form deprivation that in turn affects the resultant ionic environment of the outer and inner retinal tissues, axial and vitreal elongation as predicted by the RIDE model. Occluder removal and return to normal light conditions led to return to more normal upregulation of phototransduction, slowed growth rate, refractive recovery and apparent return towards physiological homeostasis.

Myopia (short-sightedness) is the most common visual disorder worldwide and the greatest risk factor for severe ophthalmic diseases in older individuals especially those with high (-5D) refractive errors^1^. Myopia is also a public health concern^2,3^ due to its rapid increase in prevalence (> 80% in young adults in Singapore^2^, Taiwan^4^ and China^5–7^). Indeed the global prevalence of myopia has been predicted to rise from 28% (2 billion people) in 2010 to 50% (5 billion people) in 2050^3^. The severity and early onset of myopia^7^ in many newly urbanized societies implicates both genetics and environment in its induction.

Clinical and experimental myopia share similar morphology and pathophysiology, with the hallmark characteristic being excessively large eyes with abnormal axial elongation especially of the vitreous chamber. As yet, there is no consensus as to why or how this increase in ocular volume is induced though it is well accepted that myopia is a visually driven process and that the retina, and the photoreceptors in particular, are the neural elements primarily sensitive to temporal modulation of luminance^8^. Many early animal studies exploring the aetiological mechanisms associated with the effects of abnormal light conditions on axial elongation^9,10^ were concerned with effects on scleral^11–14^ and choroidal mechanisms^13–15^. Roles for GABA^16^, glucagon^17–19^ and dopamine^20^ in refractive error development have also received extensive investigation, though few studies have explicitly considered how such theories could *physiologically* explain the large eyes that are the hallmark of clinical myopia or the source of the rapid changes in axial length in humans^21–27^ as a result of 30 min of prolonged accommodation or water drinking. In animal models of myopia,  rapid axial elongation, refractive change and altered gene expression^28^ is seen following 6 h of − 10D optical defocus in chicks or within 30mins of removal of form deprivation^29^; see Fig. 1.

**Figure 1:**
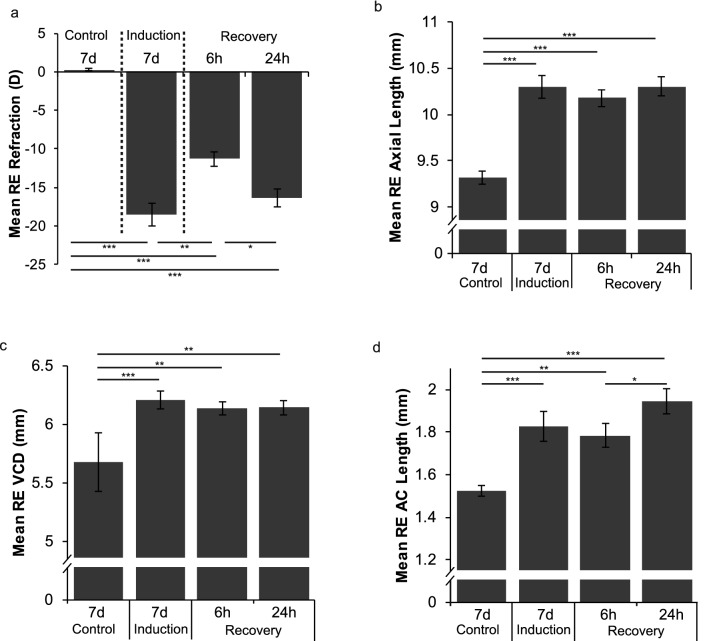
Biometric measurements of normal and form deprived (FD) chicken eyes following induction and recovery from form deprivation myopia. Chickens were visually deprived by occlusion of their right eye then given a variable number of hours of normal visual experience (T = 0 h, 6 h, and 24 h). At each timepoint (n = 8), biometric measures were taken including (**a**) refraction, (**b**) axial length, (**c**) vitreous chamber depth (VCD) and (**d**) anterior chamber (AC) length. Note: VCD is measured from the posterior surface of the lens to the fibre layer of neural retina. AC length is measured from the anterior surface of the lens to the cornea. For AL measurements of specific chicks utilised in the RNA-seq analysis, refer to Supplementary Table 9.

One such physiological model of myopia development based on very well established retinal/RPE physiology and extensive literature relating to rapid light/dark induced fluid shifts in the retina^29–33^ has been proposed by Crewther^34^ as the Retinal Ion Driven Efflux (RIDE) model of myopia. This model postulates that occlusion or acute blur perturbs phototransduction and hence slows the rate of exchange of ions and fluid between photoreceptors, sub-retinal space (SRS) and retinal pigment epithelium (RPE), and consequently attenuates the normal chloride anion driven efflux of fluid from the vitreous across the retina/RPE^30,33,35–37^ to choroid. This model is well supported by ultrastructural evidence^38^ showing edema in the retina and extensive hyperosmolar stress in the nuclei, mitochondria and basal membranes of the Retinal Pigment Epithelium (RPE)^22,29,39^. Liang et al.^29,39^ and Crewther et al.^22^ have also employed Scanning Electronmicroscopy with elemental microanalysis to demonstrate increased potassium, sodium and chloride ion concentrations across the posterior eye following form deprivation. Thus we aimed to investigate if the biochemical pathways identified by RNAseq transcriptomics during induction of FDM and short term recovery would refute or add any support for the RIDE model theory.

To date, molecular research into gene models of myopia induction have been dominated by human genome wide association studies (GWAS)^8,40–57^ and limited pathway analysis with subsequent transcriptome analyses of single differentially expressed genes (DEGs)^58–71^ or proteins^72–83^. The lack of consistent results for the above studies could largely arise due to the stability of non-profound refractions of humans in many of the GWASs and the use of differing species, ocular tissues, durations of form deprivation or hours of optical defocus and relative state of refractive compensation. However, more recent use of comparative pathway analysis techniques, and threshold free pathway analysis of multiple gene sets (ie gene set enrichment analysis (GSEA)) in particular have begun to provide more useful insights highlighting dysregulation in several fundamental biological pathways^28^. In particular, form-deprivation (FD) myopia has now been associated with molecular mechanisms of suppression of neuron structure/growth, signal transduction and inner retinal neurotransmitters associated with Cl^−^ ion transport^63,70^, while transcriptomic changes associated with early optical induction of myopic and hyperopic refractive errors also highlight altered metabolic pathways in response to lens induced myopia (LIM)^69^.

The primary aim of this study was to extend our previous transcriptome microarray study of the differentially affected genes and biochemical pathways during prolonged chick FDM and the early hours of recovery, by using next-generation RNA sequencing technology. This technique possesses greater theoretical sensitivity to further elucidate the molecular pathway underpinning the axial length changes in FDM and during recovery than microarray technology. We have aimed to assess expression of a priori identified gene sets and biological pathways associated with myopia using GSEA rather than individual differentially expressed genes (DEGs) (Reviewed in^28^). The RIDE model and previous molecular work has suggested that gene families associated with cone phototransduction and outer and inner retinal solute transport and energy metabolism, should characterize pathways likely to be associated with increased ocular growth during the induction of form-deprivation myopia (FDMI) and slower growth rates during refractive recovery (FDMR) following reintroduction of normally modulated light conditions.

## Material and methods

### Animals

Thirty-two male chicks (Leghorn/New Hampshire) were raised with unlimited food and water in a controlled environment on a 12-h light/12-h dark cycle and with the temperature maintained at 30 ± 0.5 °C. Illuminance was maintained at 183 lux during the 12 h day cycle (from 8am to 8 pm) using a 20 W halogen lamp. Twenty-four chicks were monocularly occluded on post-hatch day 5 for 7 days to induce FD myopia. The translucent polystyrene occluders were attached to the periocular feathers of the right eye. On day 12 post-hatch, occluders were removed from FD chicks at 10am and animals were either immediately sacrificed, ie 0 h recovery (n = 8), or sacrificed after 6 h (ie at 4 pm; n = 8) or 24 h (n = 8) of the normal light/dark condition (ie 10 h-light/12 h-dark condition and 2 h light to make up 24 h) following the prolonged form-deprivation. Eight separate chicks were included in the analysis as non-occluded controls at the T = 0 h timepoint.

We chose to use a separate batch of control animals rather than use the within subject contralateral fellow eyes. This decision was based on prior evidence showing binocular changes in choroidal blood flow during monocular occlusion^84,85^ and similar direction significant changes in refraction and axial length in the fellow eyes^14^. Binocular interaction effects have been suggested to account for the substantial differences in gene expression as observed by He et al.^86^ between contralateral eyes and separate controls (see also^75,87,88^, hence we have chosen to use separate control animals for these reasons. All animal work in this study was approved by the La Trobe University Animal Ethics Committee (Approval number AEC 11/68) and is in accordance with the ARVO Guidelines for Use of Animals in Research, Australian NHMRC Animal Ethics requirements and the ARRIVE guidelines^89^.

### Ocular biometrics analysis

Refractive state (dioptres (D)), vitreous chamber depth (VCD in mm), axial length (AL in mm) and anterior chamber (AC in mm) measures were collected from all animals on day 12 post-hatch and after 0 h recovery (n = 8), 6 h recovery (n = 8) and 24 h recovery (n = 8) while animals were lightly anesthetized with an intramuscular injection of a mixture of ketamine (45 mg/kg) and xylazine (4.5 mg/kg). Refraction in the experimental right eyes were determined by trained ophthalmic practitioners using retinoscopy (Keeler, Vista Diagnostic Instruments) and A-Scan ultrasonography (A-Scan III, TSL; Teknar, Inc. St Louis, USA; 7 MHz probe) was used to measure axial dimensions. Baseline biometric measures were not sought to avoid repeated potential anaesthesia effects on eye growth^67^, RNA quality and sequencing^90–93^. Chicks were only sedated prior to decapitation. Quantitative data were expressed as Means (+ /− Standard Error). Analysis of Variance (ANOVA) measuring group differences was carried out between same age controls, form-deprived and recovery eyes to determine significant changes in biometric measurements followed by post-hoc tests as required.

### RNA isolation and cDNA library preparation

Tissue samples for RNA isolation and sequencing were collected from right eyes only, from four out of eight chicks per time condition. These four chicks were chosen based on comparable axial length measurements (see Supplementary Table 13). Total RNA was isolated from posterior eye retina/RPE/choroid tissue using the Trizol method and the RNeasy mini kit (Qiagen) with the on-column DNA digest according to manufacturer’s instructions. RNA quality was measured using the Agilent 2100 Bioanalyzer (RNA 6000 Nano Kit; Agilent Technologies, Santa Clara, CA, USA). All samples had a RNA integrity number (RIN) above 8.7. RNA was also quantitated on the Qubit 2.0 Fluorometer (RNA HS kit; Invitrogen, Australia). For library preparation, RNA concentration was calculated using an average measure from the Bioanalyzer and Qubit assays. A total of 2.5 µg of mRNA was purified from total RNA using oligo (dT)-conjugated magnetic beads (Illumina, San Diego, CA, USA). The fragmented mRNA was then subjected to cDNA synthesis using the TruSeq Stranded mRNA kit (Illumina, San Diego, CA, USA) following the manufacturer’s low-sample throughput protocol. All cDNA libraries were assessed on an Agilent 2100 Bioanalyzer (DNA 1000 kit; Agilent Technologies, Santa Clara, CA, USA). Size of the final products were approximately 260 bp. cDNA libraries were quantitated using 3 methods: Bioanalyzer (DNA 1000 kit; Agilent Technologies, Santa Clara, CA, USA), Qubit (dsDNA HS assay; Invitrogen) and qPCR (GeneRead Library Quant Array; Qiagen, Germantown, MD, USA). DNA libraries were normalised to 10 nM with Tris–HCl 10 mM, pH 8.5 with 0.1% Tween 20 and pooled. Libraries were denatured with 0.1 N NaOH. 7 pM of denatured libraries were prepared for cluster generation on the Illumina cBot using the TruSeq SR Cluster Kit V3-cBot (Illumina, San Diego, CA, USA). The sequencing run involved a duel-index, single-end sequencing run of 1 × 100 cycles on an Illumina HiSeq 1500 using the TruSeq SBS Kit V3 (Illumina, San Diego, CA, USA) sequencing reagents. Average mapped counts for each sample were 6,814,421 (Supplementary Table 8). RNA-Seq data for each sample are available at the NCBI Gene Expression Omnibus (accession number GSE80327).

### Sequencing data pre-processing

Quality of the sequencing data was assessed with fastqc (https://www.bioinformatics.babraham.ac.uk/projects/fastqc/). Sequences with Q score < 10 along with adapters were removed using CutAdapt^94^ and Trimmomatic^95^. Sequence reads were mapped to the chick genome (GalGal4) using TopHat2^96,97^ and Bowtie2^98^. Htseq-counts were used to count the number of reads that uniquely mapped to a gene (Supplementary Table 10).

### Data analysis

Data were analysed to identify differentially expressed genes (DEGs) and enriched pathways in a pairwise manner using a moderated t-test following prolonged occlusion (0 h vs Control) and during the 24 h recovery period (6 h vs 0 h, 24 h vs 6 h, and 24 h vs 0 h). Additionally, DEGs and enriched pathways across the entire recovery period were identified using a moderated F-test and the Gene Set Enrichment Analysis Pearson’s correlation metric, respectively. Specific details for each step in the analysis are outlined below.

#### Differential gene expression

Data was filtered to only include genes with at least 10 counts (or 1/million) in at least 4 out of 12 samples. DEGs were calculated using EdgeR^99^ as implemented in Degust (http://degust.erc.monash.edu). We assessed for DEGs after prolonged occlusion (0 h vs Control) and during the 24 h recovery period (6 h vs 0 h, 24 h vs 6 h, and 24 h vs 0 h) using a moderated t-test with a false discovery rate (FDR) of 5%. To determine the number of DEGs over the 24 h recovery period (0 h, 6 h and 24 h), we used a moderated F-test (i.e. ANOVA) with FDR of 5%. Functional classifications of DEGs were characterised using the Protein Analysis Through Evolutionary Relationships (PANTHER) Classification System (v13.1; http://www.pantherdb.org/)^100^.

#### Pathway enrichment analysis

Broad Institute’s Gene Set Enrichment Analysis (GSEA) software was used to determine whether an a priori defined set of genes is statistically significant^101,102^ during the induction and recovery of FD. The ‘CP: Canonical Pathway' gene sets were obtained from the Molecular Signatures Database (MsigDb v6.2; http://software.broadinstitute.org/gsea/msigdb/index.jsp)^101,103^. This collection of pathways includes curated gene sets from various online pathway databases, the biomedical literature, and contributions from domain experts. The four main databases comprising this collection include KEGG (http://www.genome.jp/kegg/pathway.html), Pathway Interaction Database (http://pid.nci.nih.gov/), Reactome (http://www.reactome.org/) and the Signal Transduction Knowledge Environment (STKE)^104^. Chick Ensemble Gene IDs were mapped to human gene symbol using BioMart (Ensembl release 89; Supplementary Table 11). Where mapping resulted in no known gene, Chick Ensemble Gene IDs were mapped to human gene orthologues (Homo sapiens GRCh38.p7). The conversion of Chick IDs to human gene symbols was done as the MsigDb and associated genesets that are based on human gene annotations.

The GSEA technique involves ranking all genes within a sample dataset based on their differential expression between two experimental groups using a basic metric e.g. signal-to-noise ratio (S2N), ratio of average expression from two classes (Ratio), T-test statistic (T-test), or the Pearson correlation coefficient for quantitative studies^101,102,105^. The method then evaluates the general differences in the cumulative distribution in expression of genes in a biological pathway based on a priori knowledge of the gene’s biological function (i.e., gene sets from the Molecular Signatures Database (MSigDb)). An enrichment score (ES) for each gene set is calculated. This ES reflects the degree to which a geneset is overrepresented at the top or bottom of a ranked list of genes. The ES is calculated by walking down the ranked list of genes, increasing a running-sum statistic when a gene is in the gene set and decreasing it when it is not. The ES for each pathway reflects the maximum deviation from zero encountered in walking down the list. A normalised enrichment score (NES) is also calculated by GSEA in which differences in pathway size (i.e. geneset size) are considered, allowing for comparisons between pathways within the analysis^101,102^. In this study, the default *Signal2Noise* metric was used to determine significantly enriched pathways following prolonged occlusion (0 h vs Control) and during the 24 h recovery period (6 h vs 0 h, 24 h vs 6 h, and 24 h vs 0 h). This metric uses the difference of means scaled by the standard deviation^106^. For comparisons purposes, the Pearson’s correlation metric was used as recommended for time-series data^106^, to assess changes in gene expression associated with the entire recovery period post occluder removal (i.e., 0 h vs 6 h vs 24 h). The analysis involved 1000 gene set permutations with gene sets limited to 15–500. As recommended by The Broad Institute^106^ for exploratory studies, a FDR of 25% was used for all bioinformatic analyses.

To understand which genes contributed to the gene set’s enrichment signal, we performed leading-edge subset (LES) analysis. This analysis identifies the core genes by creating a ranked list. The genes located at the top of the ranked list are considered to be upregulated and those at the bottom of the list are downregulated^101^.

### Gene validation

As the primary focus of this study was to uncover the molecular pathway underpinnings of FD myopia, validation of the large number (> 300) of core genes identified from this study using qPCR was not feasible to assess. Instead we aimed to validate the large number of core genes using our previously published microarray study of short term refractive recovery in chick eyes following 10 days of form deprivation^70^. There is also widespread agreement in the literature that highlights good concordance in gene expression between microarray and qPCR methods^107–112^. Briefly, in our previous microarray study^70^, RNA from retina/RPE/choroid was isolated from the posterior eye cup of form deprived chicks under the same experimental parameters as described above. High quality RNA from each animal (control and experimental) was pooled in equimolar amounts by experimental condition (control (n = 5), 0 h (n = 5), 6 h (n = 5) and 24 h (n = 5) recovery time) and sent to the Australian Genome Research Facility Ltd (Walter and Eliza Hall Institute, Victoria, Australia) for microarray processing (Affymetrix, Inc). Raw data was exported as CEL files containing probe level intensities for preprocessing with Expression Console 1.1 (Affymetrix, Inc). The raw data was summarised and normalised using the Robust Multichip Average (RMA) algorithm to yield log base 2 expression values for each gene transcript. This data can be found on the GEO Database (www.ncbi.nlm.nih.gov/geo/; accession number GSE89325).

## Results

### Ocular biometrics analysis

Refractive state (dioptres (D)) was assessed using standard retinoscopy while vitreous chamber depth (VCD in mm), axial length (AL in mm) and anterior chamber depth (AC in mm) were assessed using A-scan ultrasonography (Fig. 1). Significant myopia (− 18.6D ± 1.44) was achieved after 7 days of occlusion. Six hours of visual experience induced a significant hypermetropic shift of + 7.3D, reducing the mean level of myopia in FD eyes down to -11.3D ± 0.91. Less refractive compensation was seen after 24 h of visual recovery post-occluder removal (− 16.4 ± 1.18D), presumably due to the circadian impact of the regular night period with this measurement being made 2 h after the first 12 h night/dark period (Fig. 1a).

Axial length (AL), vitreous chamber depth (VCD) and anterior chamber (AC) showed similar patterns of biometric growth consistent with previously published diurnal rhythm changes^113–115^. Prolonged occlusion increased axial length from 9.32 mm for controls to 10.30 mm (Fig. 1b), VCD from 5.65 mm to 6.21 mm (Fig. 1c) and AC (Fig. 1d) from 1.53 to 1.83 mm. In recovering eyes, AL decreased by 0.12 mm at both 6 h and 24 h, however these changes were not significantly different to AL at occluder removal (T = 0 h; *p* > 0.05). The same trend was also seen in VCD and AC at 6 h with VCD reduced by 0.08 mm and AC by 0.04 mm indicating that the excessive ocular growth response to FD is inhibited by occluder removal and that normal light conditions favour re-emmetropisation.

### Differentially expressed genes in prolonged FD and FD recovery

To identify the transcriptomic mechanisms involved in the response to, and recovery from FD, single-gene expression changes were analysed. We first assessed DEG after prolonged occlusion (i.e. 7d of FD with 0 h recovery) and during recovery (6 h vs 0 h, and 6 h vs 24 h) using EdgeR with a moderated t-test and a false discovery rate (FDR) of 5%.

**Figure 2:**
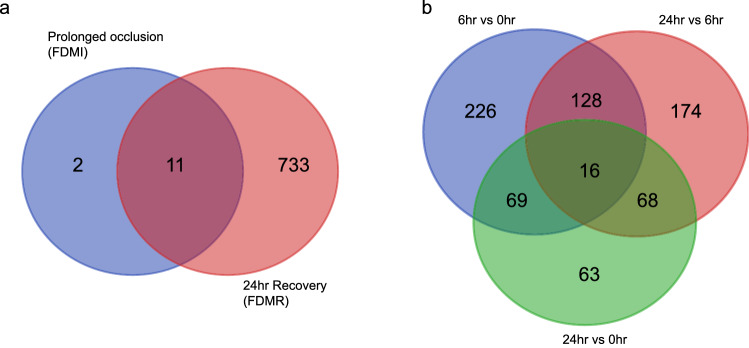
Venn diagram of overlapping and unique transcripts for all comparison time points. Significant (*p* < .05) transcripts were identified using EdgeR. (**a**) 11 of the 13 differentially expressed transcripts were common in both prolonged occlusion (7d of FD) and during the 24 h recovery period. Only 2 transcripts were uniquely expressed in response to prolonged occlusion and 733 transcripts were uniquely expressed in the 24 h recovery from FD. (**b**) There were 744 unique transcripts significantly expressed throughout the recovery period with 226 transcripts uniquely expressed after 6 h recovery compared to 0 h, 174 transcripts uniquely expressed after 24 h recovery compared to 6 h, 63 transcripts uniquely expressed after 24 h compared to 0 h and 16 transcripts common across all 3 recovery time-point comparisons.

**Figure 3:**
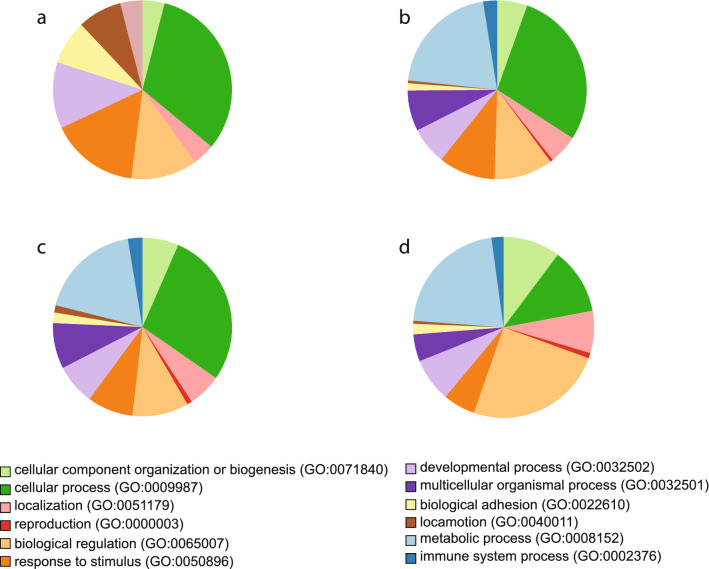
Gene Ontology (GO) classification of differentially expressed genes (DEG) after prolonged occlusion and during early recovery from form-deprivation myopia. Significant (*p* < .05) DEGs were identified using EdgeR between (**a**) No-Lens Control and prolonged form-deprivation myopia (FDM; ie 0 h recovery) (**b**) 0 h and 6 h recovery, (**c**) 6 h and 24 h recovery, and (**d**) overall recovery (0 h, 6 h and 24 h). After 7 days of FD with 0 h recovery, the DEG were mostly involved in cellular processes and response to stimulus. There are few DEGs associated with metabolic and immune system processes after prolonged occlusion compared to controls possibly indicating a new homeostatic state. However, the genes identified during recovery were mostly involved in biological regulation, cellular and metabolic processes along with the reinstatement of immune system processes. For names of individual DEGs see Supplementary Table 8.

Prolonged occlusion, relative to no-lens controls, induced expression changes in 13 genes with an FDR < 5%. BMP2, ALDH1A2, TNC, SHC4, GSN, SIK1 were down-regulated at 7d of FD relative to controls, while LOC417800, HCK, WNT9A, KIAA1199, CLEC3B, SLCO1C1 and RHOB were upregulated in the FD eye compared to controls (Supplementary Table 1). By comparison, recovery from FD identified 439 transcripts at 6 h compared to 0 h, 386 transcripts between 6 and 24 h, and 216 transcripts at 24 h compared to 0 h (Supplementary Tables 2–4). To provide a comparison with past transcriptomic studies on chick FD myopia and early recovery^63,70^ we analysed all time-points (0 h, 6 h and 24 h) using a moderated F-test which identified a total of 828 transcripts significantly differentially expressed across the 24 h period of normal day/night conditions following 7 days of form deprivation (Supplementary Table 5). The number of overlapping and unique transcripts identified is illustrated in Fig. 2 with the majority of these gene transcripts involved in cellular and metabolic processes (Fig. 3).

### Enriched pathways during prolonged FD

**Figure 4:**
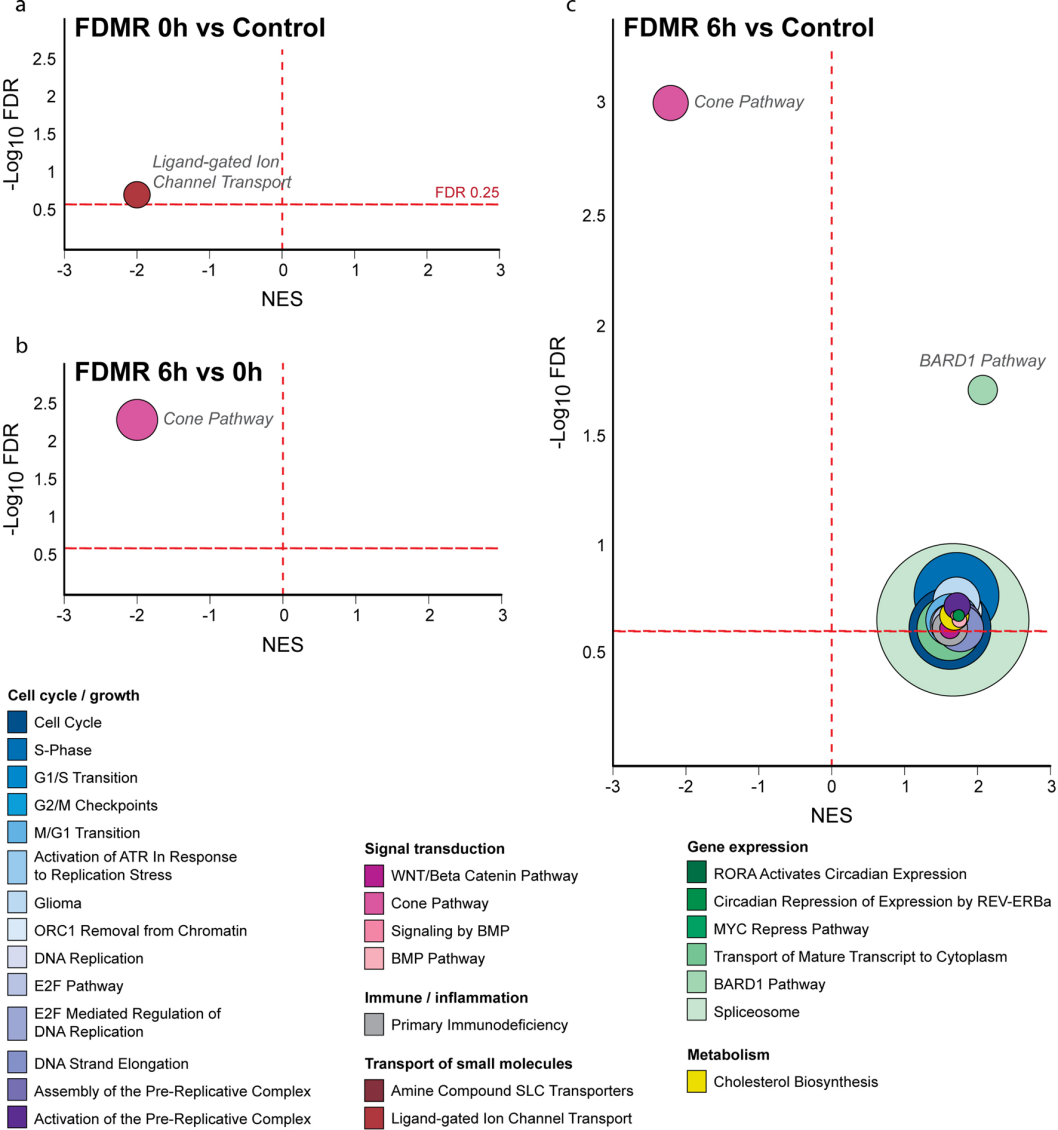
Bubble plots showing all significant pathways from the pairwise GSEA comparisons during the induction of myopia and the first 6 h of recovery from FDM. The significantly enriched pathways are visualised for (**a**) 0 h versus No-Lens Control, (**b**) 6 h versus 0 h, and (**c**) 6 h versus Control. The size of each bubble is proportional to the number of core genes within the pathway. Full details of significant pathways (including core genes driving enrichment) are provided in Supplementary Tables 1–4. NES, Normalised Enrichment Score.

Following the identification of the DEGs during induction and recovery from FD, GSEA was employed to identify changes in expression of biological pathways associated with 7 days of FD relative to normal, no lens controls. Prolonged occlusion resulted in suppression of only one pathway, ligand-gated ion channel transport (Table 1; Fig. 4). The leading-edge subset (i.e., core genes) of this pathway implicate suppression of several GABA ionotropic receptors (ie GABA_A_ and GABA_A_-rho receptors (previously known as the GABA_C_ receptors)) and glycine receptors. Such receptors are associated with Cl^−^ channels^116,117^ and would be expected to play a role in transretinal fluid movement from vitreous to choroid^30,31,34^. The core genes identified in the ligand gated pathway show good concordance with gene expression from our previous microarray study using the same experimental paradigm (Supplementary Table 12).

**Table 1:** Biological Pathways Significantly Altered during FDMI. GSEA reveals 1 Reactome pathway significantly suppressed after prolonged occluder wear. *ES, enrichment score; NES, normalised enrichment score; FDR, false discovery rate.*

Pathway	Database	Total # Genes Measured	# Genes contributing to ES	Leading edge subset genes	ES	NES	FDR
Ligand-gated Ion Channel Transport	Reactome	16	9	GABRR2, GABRA6, GABRG2, GLRA3, GABRA2, GLRA2, GABRA5, GABRA1, GABRG3	− 0.68	− 1.96	.19

### Enriched pathways during recovery from FDM

**Figure 5:**
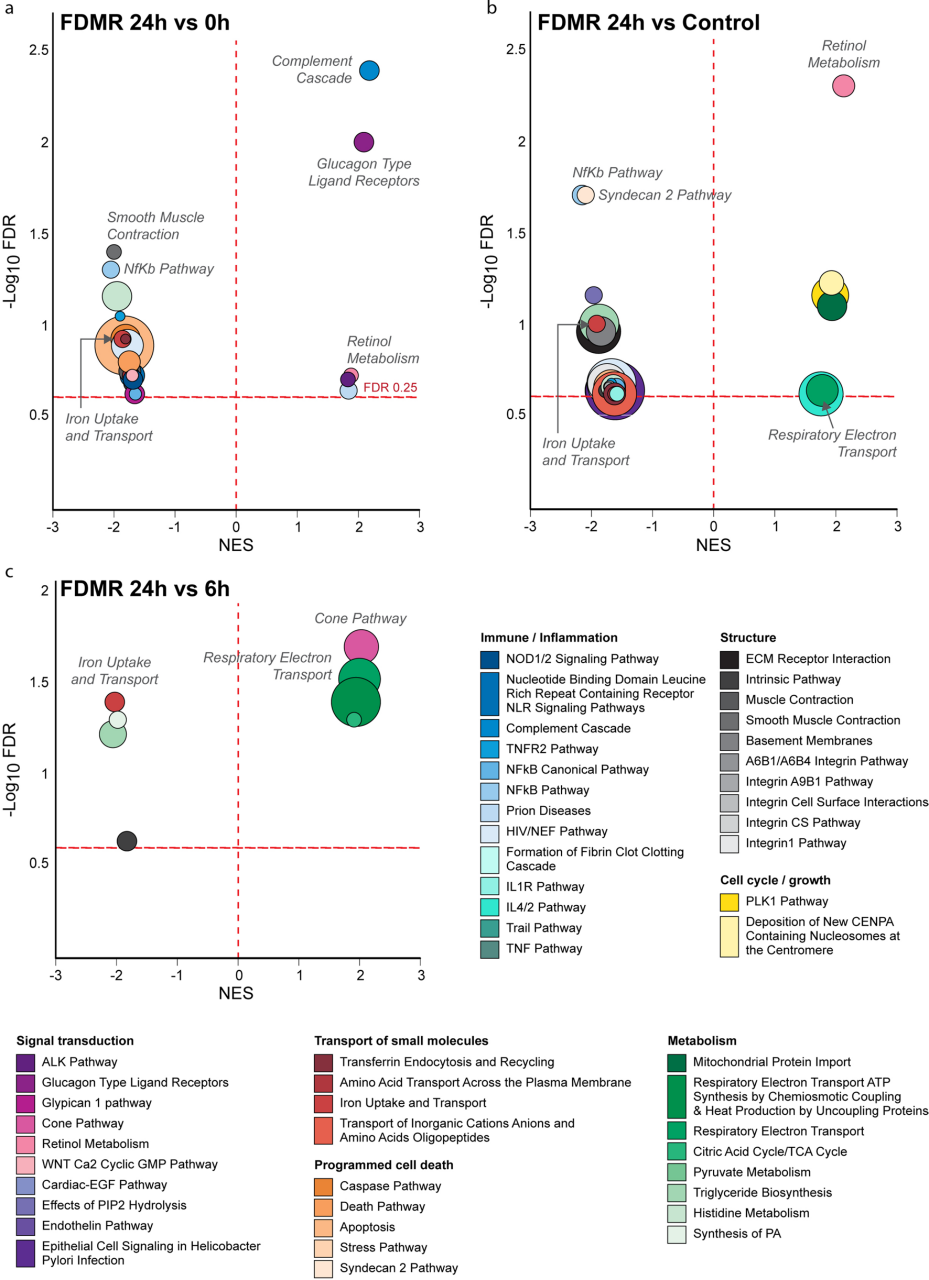
Bubble plots showing all significant pathways from the pairwise GSEA comparisons following 24 h of recovery from FDM. The significantly enriched pathways are visualised for (**a**) 24 h versus 0 h, (**b**) 24 h versus Control and (**c**) 24 h versus 6 h. The size of each bubble is proportional to the number of core genes within the pathway. Full details of significant pathways (including core genes driving enrichment) are provided in Supplementary Tables 1–4. NES, Normalised Enrichment Score.

**Figure 6:**
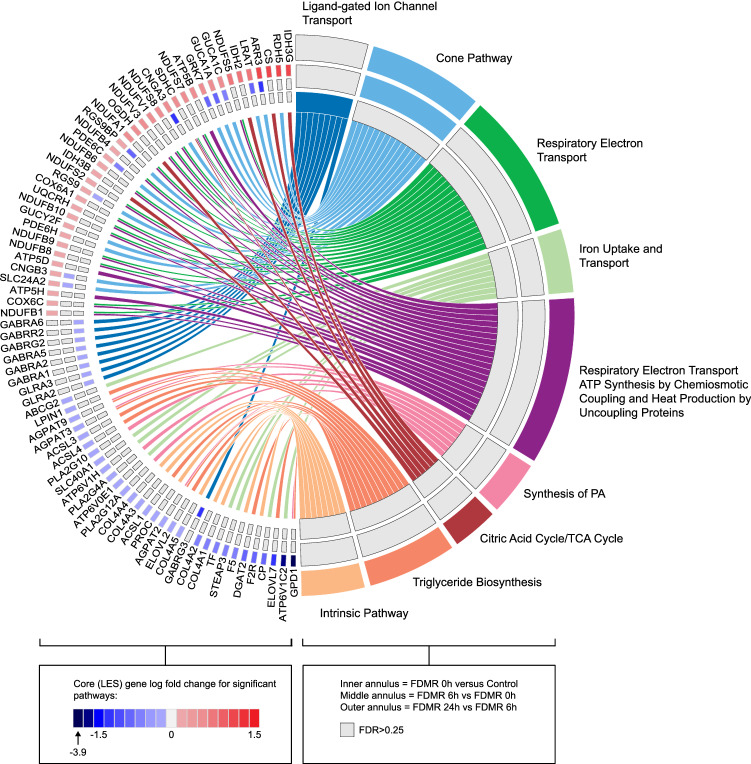
Chord diagram showing the core genes of significant pathways in FDMR 0 h versus control, FDMR 6 h versus 0 h and FDMR 24 h versus 6 h as different annuli of genes. Significant pathways are shown on the right, and the fold change of core genes is shown on the left. Left–right connections indicate gene membership in a pathway’s leading-edge subset. PA, phosphatidic acid. Note: Image constructed using GOplot R package^157^.

Pathway changes during recovery period were evaluated using the *Signal2Noise* metric by comparing gene expression changes between each recovery time-point (6 h vs 0 h, 24 h vs 6 h, and 24 h vs 0 h). The most significantly altered pathways identified in this analysis implicate early cone phototransduction, mitochondrial bioenergetics, retinol metabolism, complement and glucagon in early recovery from FD and in the myopia pathophysiology (Fig. 6). As expected, the data in Fig. 4 suggests that cone receptor phototransduction is still suppressed after only 6 h normal visual experience, i.e. cone phototransduction pathway has not totally recovered its control levels during the first 6 h of visual experience and recovery after occluder removal. An earlier electrophysiological study^38^ also found reduced cone sensitivity immediately following prolonged FD which would support such gene pathway suppression in early (6 h) and prolonged recovery (24 h). Indeed, our results comparing gene expression changes between each recovery time-point of 6 h and 24 h also indicate that the pathways underlying cone phototransduction were significantly upregulated when evaluated using the *Signal2Noise* metric as demonstrated in Figs. 4 and 5 and in Table 2. Interestingly, while transcriptomic changes in mitochondrial metabolism are absent during the first 6 h of recovery, increased mitochondrial metabolism is observed over the next 18 h (Fig. 5; Table 2) after a 1 day/night cycle has been completed. These changes occur in parallel to the increase in expression of the high-energy consuming Na^+^-Ca^2+^-K^+^ exchanger (NCKX2, also known as SLC24A2), which is characteristic of the dark-adapted retina^118^ (Fig. 6).

**Table 2:** Enriched Pathways Significantly Altered during Recovery from Form-deprivation (FDMR). GSEA was used to identify pathways (KEGG, Reactome, STKE and PID) significantly altered between recovery time-points 6 h and 24 h, compared to 0 h using the *Signal2Noise* metric. Gene expression changes at 6 h compared to 0 h were associated with suppression in cone receptor phototransduction. The period between 6 and 24 h post occluder removal was mainly associated with changes in mitochondrial metabolism and other metabolic pathways. Pathways significantly altered after 24 h recovery compared to 0 h involves a range of cellular processes. *ES, enrichment score; NES, normalised enrichment score; FDR, false discovery rate.*

Pathway	Database	Total # Genes Measured	# Genes contributing to ES	Genes contributing to ES	ES	NES	FDR
**FDMR_6 h versus FDMR_0 h**							
Cone Pathway	PID	18	14	RGS9, CNGB3, SLC24A2, PDE6C, GUCA1C, RGS9BP, LRAT, GUCA1A, GRK7, ARR3, CNGA3	− 0.79	− 2.07	0.005
**FDMR_24 h versus FDMR_6 h**							
Cone Pathway	PID	18	14	ARR3, LRAT, GUCA1C, GUCA1A, GRK7, CNGA3, RDH5, RGS9BP, PDE6C, RGS9, GUCY2F, PDE6H, CNGB3, SLC24A2	0.74	2.03	0.02
Respiratory Electron Transport^≠^	Reactome	46	17	NDUFS5, NDUFS7, NDUFA1, NDUFS8, NDUFV1, NDUFB4, SDHC, NDUFB6, COX6A1, UQCRH, NDUFV3, NDUFB10, NDUFS2, NDUFB9, NDUFB8, COX6C, NDUFB1	0.58	2	0.03
Iron Uptake and Transport	Reactome	28	8	ABCG2, ATP6V0E1, SLC40A1, TF, ATP6V1H, STEAP3, CP, ATP6V1C2	− 0.61	− 2.03	0.04
Respiratory Electron Transport ATP Synthesis by Chemiosmotic Coupling and Heat Production by Uncoupling Proteins^≠^	Reactome	55	20	NDUFS5, NDUFS7, ATP5B, NDUFA1, NDUFS8, NDUFV1, NDUFB4, SDHC, NDUFB6, COX6A1, UQCRH, NDUFV3, NDUFB10, NDUFS2, NDUFB9, NDUFB8, ATP5D, ATP5H, COX6C, NDUFB1	0.55	1.94	0.04
Synthesis of PA	Reactome	17	7	PLA2G10, AGPAT9, AGPAT3, AGPAT2, PLA2G4A, PLA2G12A, GPD1	− 0.69	− 1.98	0.05
Citric Acid Cycle/TCA Cycle^≠^	Reactome	18	6	IDH2, OGDH, IDH3G, CS, SDHC, IDH3B	0.69	1.91	0.05
Triglyceride Biosynthesis^≠^	Reactome	31	11	DGAT2, AGPAT9, ACSL1, ELOVL2, GPD1, ACSL4, ACSL3, ELOVL7, LPIN1, AGPAT2, AGPAT3	− 0.6	− 2.06	0.06
Intrinsic Pathway	Biocarta	15	8	PROC, COL4A2, COL4A1, COL4A4, COL4A3, F2R, COL4A5, F5	− 0.64	− 1.83	0.23
**FDMR_24 h versus FDMR_0 h**							
Complement Cascade^≠^	Reactome	16	8	C1QB, CRP, C1QA, C7, PROS1, CFI, MASP1, C2	0.78	2.18	0.004
Glucagon Type Ligand Receptors^≠^	Reactome	21	8	GLP1R, GHRHR, ADCYAP1, GNGT2, GHRH, GCG, VIP, GNG11	0.69	2.09	0.01
Smooth Muscle Contraction	Reactome	16	6	ACTA2, CALD1, LMOD1, MYH11, VCL, MYLK	− 0.72	− 2	0.04
NFkB Pathway^≠^	Biocarta	18	7	NFKBIA, IL1R1, TNFAIP3, RIPK1, TAB1, TNFRSF1B, TNFRSF1A	− 0.72	− 2.05	0.05
Histidine Metabolism	KEGG	20	12	ALDH1A3, HAL, MAOB, UROC1, MAOA, METTL6, LCMT2, TRMT11, HNMT, ASPA, LCMT1, CNDP1	− 0.65	− 1.95	0.07
TNFR2 Pathway^≠^	Biocarta	15	4	NFKBIA, TNFAIP3, RIPK1, TNFRSF1B	− 0.71	− 1.9	0.09
Transferrin Endocytosis and Recycling	Reactome	17	4	STEAP3, TF, ATP6V1H, ATP6V1C2	− 0.64	− 1.81	0.12
Iron Uptake and Transport	Reactome	28	7	STEAP3, SLC46A1, TF, ATP6V1H, CP, ABCG2, ATP6V1C2	− 0.58	− 1.86	0.12
Caspase Pathway^≠^	PID	42	12	CASP9, DFFB, ACTA1, DFFA, GSN, CRADD, CASP10, TRADD, ARHGDIB, CASP2, RIPK1, TNFRSF1A	− 0.52	− 1.81	0.12
Apoptosis^≠^	KEGG	59	24	NTRK1, DFFB, DFFA, IL1R1, TRADD, ENDOD1, TNFRSF10B, CFLAR, IL1RAP, PIK3CB, IRAK4, NGF, TNFRSF1A, CASP9, NFKBIA, PPP3CA, PPP3R1, CASP10, CASP3, PRKAR2A, CAPN2, TNFSF10, RIPK1, CAPN1	− 0.49	− 1.83	0.13
HIV/NEF Pathway	PID	29	13	DFFB, DFFA, CRADD, TRADD, CFLAR, TNFRSF1A, CASP9, NFKBIA, CASP3, CASP2, RIPK1, CD247, MAP3K5	− 0.55	− 1.78	0.13
HIV/NEF Pathway^≠^	Biocarta	47	13	DFFB, DFFA, GSN, CRADD, TRADD, PRKCD, TNFRSF1B, TNFRSF1A, CASP9, NFKBIA, ARHGDIB, CASP2, RIPK1	− 0.51	− 1.82	0.13
Death Pathway^≠^	Biocarta	27	9	CASP9, NFKBIA, DFFB, DFFA, CASP10, TRADD, TNFSF10, TNFRSF10B, RIPK1	− 0.55	− 1.75	0.16
Muscle Contraction	Reactome	30	9	ACTA2, DES, MYL1, CALD1, TNNC1, LMOD1, MYH11, VCL, MYLK	− 0.54	− 1.74	0.18
Nucleotide Binding Domain Leucine Rich Repeat Containing Receptor NLR Signaling Pathways	Reactome	31	10	PANX1, CASP9, P2RX7, MAPK11, RIPK2, CARD9, CASP2, TNFAIP3, NOD1, TAB1	− 0.53	− 1.7	0.19
Amino Acid Transport Across the Plasma Membrane	Reactome	19	7	SLC7A6, SLC7A9, SLC6A14, SLC16A10, SLC7A11, SLC38A2, SLC7A2	− 0.59	− 1.72	0.19
WNT Ca2 Cyclic GMP Pathway	STKE	17	5	TF, ITPR2, PDE6C, PDE6B, ITPR3	− 0.6	− 1.7	0.19
Retinol Metabolism^≠^	KEGG	17	6	RDH8, ALDH1A2, ALDH1A1, RDH5, DHRS3, BCMO1	0.67	1.88	0.19
NOD1/2 Signaling Pathway	Reactome	23	8	CASP9, MAPK11, RIPK2, CARD9, CASP2, TNFAIP3, NOD1, TAB1	− 0.56	− 1.69	0.2
ALK Pathway	Biocarta	26	6	BMP4, NPPB, FZD1, BMP2, SMAD6, BMP7	0.58	1.83	0.2
Intrinsic Pathway	Biocarta	15	7	F10, COL4A2, COL4A1, COL4A4, COL4A3, F2R, F5	− 0.63	− 1.7	0.21
Prion Diseases^≠^	KEGG	23	7	C1QB, C1QA, STIP1, IL6, NOTCH1, C7, HSPA5	0.62	1.84	0.23
Glypican 1 pathway	PID	23	8	LYN, HCK, TGFB2, YES1, FLT1, SRC, TGFB3, FGF2	− 0.54	− 1.66	0.24
NFkB Canonical Pathway	PID	18	5	NFKBIA, RIPK2, TNFAIP3, BCL10, TNFRSF1A	− 0.6	− 1.65	0.24

Note: pairwise comparisons with control animals (ie 6 h vs control and 12 h vs control) can be found in Supplementary Table 6.

**NOTE:** + NES = expression upregulated;—NES = expression downregulated; ^≠^present in GSEA using Pearson’s correlation metric (Supplementary Table 7); *present in previous data^70^.

To determine if the pathways identified during FD recovery using the *Signal2Noise* metric produced similar pathway findings to the Pearson’s correlation metric, the data was then analysed (using GSEA) for pathways correlated to recovery time (0 h, 6 h, 24 h). Twenty-nine pathways were found to be significantly altered during the 24 h recovery period with the top 3 pathways involved in the complement cascade, mitochondrial energy metabolism and retinol metabolism (Supplementary Table 7). Other pathways identified by Pearson’s correlation have roles in immunity, lipid metabolism, smooth muscle contraction, apoptosis, protein metabolism, signal transduction, golgi transport, extracellular matrix (ECM), hemostasis and disease. Indeed, the findings from both the *Signal2Noise* analysis and Pearson’s correlation suggests that these metrics can produce comparable results.

## Discussion

This study has utilized pathway enrichment analysis (GSEA) to identify biological pathways shown by RNA seq to be involved in profound myopia induced by 7 days of FD and during the first 24 h of early recovery in a normal modulated light environment. Our findings raise intriguing questions regarding the temporal interaction of transcriptome changes and previously described ionic, physiological and morphological changes associated with induction and recovery from FDM^22,29^ and theoretical models of myopia.

### GABA and glycine mediated chloride ion transport pathways significantly altered in profound FDM

Consistent with the RIDE model, GSEA identified suppression of the GABA- and glycine-mediated chloride ion transport pathway as the only significantly altered biochemical pathway associated with 7 days of excessive ocular growth, thinning of the retina and choroid and altered ion distribution patterns accompanying profound refractive myopia^29^. The fact that the only pathway showing significant change after 7 days of FDMI was the chloride ion transport pathway is particularly important to understanding development of overly large volume eyes, which is the hallmark sign of myopia. As extensively studied since the early 70s^119^ and Reviewed in^120^, chloride channels reside in all plasma membranes and most intracellular organelles and are fundamental to all cell volume regulation, transepithelial transport and regulation of electrical excitability in general. Hence suppression of the Cl- channel pathways across the entire retina/RPE/choroid examined with RNAseq provides an explanation for the ultrastructurally described dehydration seen in FDM tissue in profoundly myopic eyes^22,29,39^.

### FDM recovery pathway changes – cone phototransduction first

By comparison with the stable conditions during prolonged occlusion, GSEA of RNAseq data showed rapid upregulation of many cellular processes but particularly upregulation of cone phototransduction and BARD1 expression following occluder removal and reintroduction of only 6 h of normally modulated light conditions. Within 24 h post occlusion, the new molecular state of retina/RPE/choroidal tissue was characterized by further upregulation of cone phototransduction consistent with the predictions of the RIDE model^34^. Our RNAseq findings also validate previous electrophysiological finding of cone photoreceptor sensitivity^38^ as well as our previous transcriptome work in chick using FDM^70^, and optical defocus (+ 10D and -10D) lenses^69,83^.

### Upregulation of BARD1 at 6 h recovery

Upregulation of the BARD1 pathway in the first 6 h of normal light conditions highlights that prolonged occlusion, profound myopia and the morphologically thinned choroid is persistently physiologically stressful to cellular function^121,122^. Over the last few years the BARD1 pathway has been reported to play a central role in the control of the cell cycle in response to DNA damage in many diseases and its regulation has been shown to mediate the formation of 'Lys-6′-linked polyubiquitin chains and coordinate a diverse range of cellular pathways such as DNA damage repair, histone ubiquitination and transcriptional regulation to maintain genomic stability^121–123^. The presence of BARD1 post profound FDM may be an early indicator of the types of cellular damage observed in human and animal myopia, especially with regard to thinning of the choroid and retina. This may explain why high myopia is a risk factor for secondary ophthalmic diseases later in life.

### Implications of suppressed ligand-gated chloride channel pathways for ocular growth

Significant suppression of the ligand gated chloride channel pathway following prolonged FDM is compatible with the notion that increases in axial length and thinning of the retina and choroid in response to FD occur concurrently with changes in the distribution of physiologically important ions including chloride, sodium, potassium and calcium across the posterior eye^22^. In a review of Ion Channels of the RPE, Wimmers et al.^33^ similarly to Gallemore et al.^30^, and Crewther^34^, have highlighted the role of Cl^−^ and K^+^ ions as the key drivers of transepithelial water transport and volume regulation in the retina and the interaction of the photoreceptors and the RPE. The established role of GABA and glycine in retinal third order neuronal transmission and in Cl^−^ transport^116,117^ would also suggest that hydrated Cl^−^ ions could play a particularly important role in axial growth during FD in animals^69,70,83^ and human myopia^8^. Indeed suppression of the GABA and glycine ligand-gated chloride ion-transport pathway in response to FD (Table 1) is also consistent with previous reports of significantly reduced retinal concentrations of GABA in chick FDM^16,124^ and GABA signal following abnormal axial growth in FD guinea pigs^71^. A number of pharmacological studies have also utilized GABA antagonists (e.g. TPMPA) to inhibit the response to FD by inhibiting axial elongation and vitreous chamber depth^16,125–128^. Downregulation of GABA and glycine in retinal tissue has also been seen in normally growing chicks over a 48 h period^129^, whereas the same study observed an increase in GABA signaling proteins in the same 6–48 h period when chicks wore negative lens wear and during which refractive compensation was achieved^129^.

### Ionotrophic GABA receptor distribution in the posterior eye

Interestingly GABA receptors, that play such an important role in major third order neuronal transmission^130^, are also found in abundance on RPE cells^131,132^ suggesting that suppression of such ligand-gated chloride channels in RPE and most retinal cells^133^ during FD would be expected to reduce the transretinal fluid efflux towards choroid and result in rapid increase of fluid in the vitreous and axial elongation. Furthermore, as the majority of GABA receptors identified in Table 1 are ionotropic, the role of ion homeostasis, particularly K^+^ and Cl^−^, in the development of axial elongation and myopia cannot be ignored. This is particularly so given the substantial evidence that fluid flow across the RPE is related to the ionic environment of the retina/RPE/choroid^30^. Spatial distribution of ions using X-ray microanalysis has revealed significant differences in the distribution of ions of phototransduction (Na, K, Cl, Mg and Ca) as well as other physiologically relevant ions (PO_4_^3−^ and SO_4_^2−^) known to be important for cellular functioning and structural integrity^22^. Taken together, these findings offer strong support for the role of light, cone phototransduction, RPE mechanisms, ion regulation and neurotransmission in driving myopia development and in no way refute the predictions of the RIDE model^34^.

### Metabolic recovery following reintroduction of temporally modulated light

Recovery from refractive myopia and reduced axial, vitreal and anterior chamber growth rates seen during the first 24 h following FD removal appear to be associated with both cone-dominated phototransduction and increased mitochondrial metabolism, especially at 6 h and 24 h post FD. These findings are consistent with our earlier FD microarray study^70^ and RNAseq study of refractively compensated negative lens-induced myopia in chick^69,134^. Interestingly, mitochondrial respiratory electron transport chain genes have also been identified in a human study of genetic myopia^135^, highlighting the important contribution of mitochondrial bioenergetics and the phototransduction cascade to ocular function and myopia development. This data is also in line with previous ultrastructural evidence of abnormal photoreceptor elongation, loss of mitochondrial integrity^29,39,136^, and oxidative stress^70^ during FD and subsequent reversal of most morphological changes in the 48 h following occluder removal in chick^29,137,138^. Indeed, our earlier microarray study^70^ indicated that mitochondrial respiratory complex 1 and 3 genes and mitochondrial reactive oxygen species (mROS)^139^ are upregulated during FDMI. The release of mROS has previously been suggested to be a response to cellular stress while coincidently acting as a signalling molecule to facilitate cellular adaptation to this stress^140^. In fact, mROS may upregulate cone photoreceptor pathways and hence neurotransmission in inner retina^141^, chloride transport and homeostasis^142^ which are the main processes we have reported previously following termination of occlusion and re-established normal light conditions^22,29^. Thus, the increase in mitochondrial electron transport chain mRNA seen 24 h after occluder removal (Fig. 5; Table 2) fits with the idea that the dark-adapted (low temporal luminance modulation) retinae require ~ 20% more metabolic activity than the same light-adapted retina^143^. This is not unexpected given that the FD retina has previously been shown to be in a pseudo dark-adapted state^37^ and associated with the re-accumulation of K^+^ in the subretinal space and exclusion of fluid under conditions of low temporal luminance modulation^22^. Importantly, the ATPase mechanism of the RPE apical surface is electrogenic and modulates the transepithelial potential which is closely related to the control of fluid flow across the RPE and so would be expected to be upregulated during form deprivation, lower in the first 6 h of normal light and then upregulated again during the ensuing night^34,144^.

### Immune pathways in recovery

The presence of immune related pathways in the recovery from the physiologically stressful FD condition and the upregulation of the BARD1 pathway suggest a role for the immune system in refractive compensation. Such an association has recently appeared in regards to the relation of clinical blood counts as measures of immune responses and inflammation and high myopia^145–147^. The identification of the complement pathway during recovery from FD is consistent with our earlier transcriptomic studies in chick^70,134^ and support previous well-described links between mitochondrial bioenergetics and the complement system in the body and in the eye^148–150^ and in many neurodegenerative diseases such as Alzheimer’s disease (AD), Parkinson’s disease (PD), and Amyotrophic Lateral Sclerosis (ALS)^151^.

Complement factors have also been associated with previous reports of human and animal refractive errors^70,134,152,153^ and should be expected in animal models of FD given the severe choroidal thinning and associated ultrastructural and ionic changes seen in FD eyes post occlusion^22,29^. These changes have also been demonstrated in choriocapillaris fenestration number, choroidal blood vessel permeability in chicks recovering from FDM^154^ and seen in oncosis^155,156^. The previously described increase in extravascular space edema^22,29^ and lymphatic vessel permeability^154^ immediately post-occlusion also supports our results here as well as the recent FD transcriptomics^70^. What was not expected is the involvement of other immune pathways besides complement in FD recovery.

### Limitations

Lastly, while our decision to use retina/RPE/choroid may be considered a limitation, we contend that investigation of the combined changes in expression of genes within the entire biological network of the eye is necessary to understand the photoreceptor induced temporal changes in form deprivation myopia seen in animal models. Indeed, when evaluated experimentally in combination with systematic system neuroscience review techniques, the impact of using inner and outer retina, RPE and choroid in large scale genomic and proteomic studies supports the idea that similar biological mechanisms are associated with FD and lens induced defocus, regardless of the varying combinations of tissues used^28^. Indeed, Fig. 5 illustrates the commonality of the biological processes that underlie the adaptive responses to environmental manipulation of temporal modulation of luminance information across the multiple tissue types of the posterior eye. Thus, we argue that the use of multiple tissues is not an impediment to our GSEA-based interpretation as evidenced by the robustness of our current findings with previous research in human and animals. Furthermore, GSEA has proven a useful tool in identifying significantly enriched biological pathways in large-scale ‘omic’ studies. However we also acknowledge that large-scale discovery-driven studies may produce false positives. Additionally the limited gene sets and the redundancy in the databases currently available in the Molecular Signature database may affect future interpretations of this data. Future studies may benefit from using a wider range of databases as well as re-analysing data periodically to account for updated gene and pathway information. Additionally, we have not considered the role of circadian rhythms of ocular refraction and gene expression, particularly with regard to the differences in refractive normalization seen at 6 h compared to 24 h recovery from FD. We do not have a circadian matched control for our 6 h recovery group and acknowledge that this may be a limitation in our study. Indeed, 2 circadian-related pathways (*RORA Activates Circadian Expression* and *Circadian Repression of Expression by REV-ERBa*) were highly expressed at 6 h recovery compared to the no lens control however this result should be taken with the understanding that the no lens control is not a circadian matched control for the 6 h recovery timepoint. As such, we cannot conclude if circadian rhythms have an influence on refractive development and myopia. However, our no lens controls, 7 days induction, and 24 h recovery groups all experienced the same day/night patterns with tissue collected at approximately 10am. No circadian effects were highlighted suggesting that circadian rhythms effects on gene expression will be minimal and as such, may not be a factor in refractive development and myopia. Further studies should incorporate circadian matched controls to minimise circadian rhythm effects on gene expression.

## Conclusions

We conclude that axial myopia is an adaptive response to the environmental perturbation of the visual signal cascade (i.e. Phototransduction). This perturbation in phototransduction consequently affects the ionic environment of the photoreceptors, subretinal space and inner retina as predicted by the RIDE model via GABA and glycine pathway signalling and consequent effects on ligand-gated chloride channels and their role in transretinal fluid flow. Our identification of the different mitochondrial bioenergetic profiles observed between FDMI and FDMR could be considered as a potential molecular hallmark of the myopia condition. Most importantly our findings are also consistent with the predictions of the theoretical implications of the RIDE hypothesis highlighting the importance of light driven osmoadaptive pathways, enhanced mitochondrial bioenergetics and cellular immune responses, during the development of FD myopia and during refractive recovery.

## Supplementary Information

Supplementary Information

## Data Availability

The datasets generated during the current study are available in the Gene Expression Omnibus (GEO) repository (https://www.ncbi.nlm.nih.gov/geo/; Accession # GSE80327).
